# Changrui enema inhibits inflammation-induced angiogenesis in acute radiation proctitis by regulating NF-κB and VEGF ^1^

**DOI:** 10.1590/s0102-865020200050000002

**Published:** 2020-07-06

**Authors:** Jinsheng Gao, Yousong Li, Xi Yang, Min Hu, Jie Xu, Lin Cheng, Kaiqi Cao, Likun Liu, Xixing Wang

**Affiliations:** I MD, Associate Professor, Department of Oncology, Shanxi Province Research Institute of Traditional Chinese Medicine, Taiyuan, China. Conception and design of the study, analysis and interpretation of data, manuscript writing, final approval.; II MD, Associate Professor, Department of Traditional Chinese Medicine, Shanxi Bethune Hospital, Taiyuan, China. Analysis and interpretation of data.; III MD, Postdoctor, Longhua Hospital, Shanghai University of Traditional Chinese Medicine, Shanghai, China. Analysis and interpretation of data.; IV PhD, Department of Oncology, Shanxi Province Research Institute of Traditional Chinese Medicine, Taiyuan, China. Technical procedures, analysis and interpretation of data.; V PhD, Department of Oncology, Shanxi Province Research Institute of Traditional Chinese Medicine, Taiyuan, China. Manuscript writing.; VI Full Professor, Department of Oncology, Shanxi Province Research Institute of Traditional Chinese Medicine, Taiyuan, China. Conception and design of the study.; VII Full Professor, Department of Oncology, Shanxi Province Research Institute of Traditional Chinese Medicine, Taiyuan, China. Conception and design of the study, provision of reagents, materials and analysis tools.

**Keywords:** Proctitis, Enema, Inflammation, Mice

## Abstract

**Purpose:**

Changrui enema, a traditional Chinese medicine prescription, is used as a supplementary treatment for acute radiation proctitis (ARP). Herein we explored the inhibition effects of Changrui enema on NF-κB and VEGF in ARP mice.

**Methods:**

A total of 120 C57BL/6 mice were divided randomly into normal mice group, ARP mice group, western medicine enema group (dexamethasone combined with gentamicin), and Changrui enema group. ARP mice were established by pelvic local irradiation. The expression of IL-1β, NF-κB, VEGF, AQP1, AQP3, p-ERK1/2 and p-JNK was determined by immunohistochemistry or western blot.

**Results:**

The study firstly found that Changrui enema alleviated ARP mice. The expression of IL-1β, NF-κB, VEGF, AQP1 and p-ERK1/2 was increased in ARP mice, and was reserved by Changrui enema. However, the expression of AQP3 and p-JNK was decreased in ARP mice, and was up-regulated by Changrui enema.

**Conclusions:**

Changrui enema is an effective treatment with fewer side effects for ARP. The mechanism of Changrui enema may be related to the inhibition of inflammation-induced angiogenesis. Changrui enema inhibits IL-1β and NF-κB expression as well as VEGF expression. Interestingly, AQP1 promotes angiogenesis, while AQP3 inhibits inflammation. Changrui enema probably inhibits AQP1 expression by down-regulating p-ERK1/2, and improves AQP3 expression by up-regulating p-JNK.

## Introduction

Radiation proctitis is the most common radiotherapy side effect^1^ of abdominal and pelvic malignant tumors, such as colorectal cancer, ovarian cancer, prostate cancer and bladder cancer etc. The acute and chronic radiation proctitis incidence is 50%-75% and 5%, respectively^2^ . Patients with radiation proctitis mainly suffered from abdominal pain, diarrhea, excretion of mucus or blood and so on. Therefore, radiation proctitis results in poor quality of life, radiotherapy failure, and unfavorable prognosis of the patients. Unfortunately, radiation proctitis therapies for radiation proctitis are less effective and have more side effects.

Traditional Chinese medicine (TCM) has been used to treat radiation proctitis. Enema with traditional Chinese medicine offers a safe, efficacious and low-toxicity treatment for ulcerative colitis^3^ and radiation proctitis^4^ . The enema is more beneficial to intestinal absorption compared with oral administration. Changrui enema, a traditional Chinese medicine compound prescription patent (Chinese patent ZL200810054678.2), is used as a supplementary treatment for ARP and is undergoing phase II clinical trials in China. Changrui enema contains 7 herbs ( Table 1 ), including 30g Sanguisorba officinalis L., 15g Agrimonia pilosa Ledeb., 6g Panax pseudoginseng, 30g Bletilla striata, 12g Colla corii asini, 10g Rheum officinale Baill., and 6g Acacia catechu. These herbs are often used for hemostasis, detoxification, clearing heat and relieving pain, which are all associated with radiation proctitis. It has been found that these herbs have inhibitory effects on inflammation or angiogenesis. Sanguisorba officinalis L. inhibits human breast cancer cells by inducing apoptosis and inhibiting angiogenesis^5^ . Agrimonia pilosa Ledeb. extract attenuates the activation of macrophages, basophils, and inhibits airway inflammation of the ovalbumin-induced mouse^6^ . Panax pseudoginseng extract acts as an anti-inflammatory, antioxidant, hemostatic and so on^7^ . Bletilla striata extract has anti-inflammation, anti-oxidation and antiulcer effects etc.^8^ . Colla corii asini (donkey-hide gelatin) is a famous traditional Chinese medicine, with known hematopoiesis as well as radio-protection^9^ . Emodin, an anthraquinone derivative extracted from Rheum officinale Baill., may effectively delay the progression of airway inflammation in a murine model of asthma by inhibiting NF-κB activation^10^ . The methanolic bark extract of Acacia catechu Willd may be beneficial in ameliorating the benzo(a)pyrene-induced oxidative stress, inflammation, and apoptosis in the lungs of mice^11^ .

**Table 1:** Herb medicines of Changrui enema.

Traditional Chinese medicine	Used part	Chinese Name	Proportion	Traditional effects
Sanguisorba officinalis L.	Root	Diyu	30g	Hemostasis and relieving pain
Agrimonia pilosa Ledeb.	Stem and leaf	Xianhecao	15g	Hemostasis and detoxification
Panax pseudoginseng	Root	Sanqi	6g	Hemostasis and relieving pain
Bletilla striata	Tuber	Baiji	30g	Hemostasis and astringent
Colla corii asini	Donkey-hide gelatin	Ejiao	12g	Hemostasis and hematopoiesis
Rheum officinale Baill.	Root and stem	Daihuang	10g	Clearing heat and detoxification
Acacia catechu	Peeled branch and trunk	Ercha	6g	Hemostasis and clearing heat

We found that Changrui enema effectively relieved the ARP symptoms including hematochezia, diarrhea, and abdominal pain in clinical application. The pathological results under microscopy exhibited significant hyperemia and edema in rectal mucous of ARP patient. Other studies^12^ have found that angiogenesis in radiation proctitis may be an important factor related with inflammation. Nuclear factor-kappa B (NF-κB) is a heterodimeric transcription factor that plays a key role in inflammatory^13^ . NF-κB promotes the transcription of IL-1β^14^ . NF-κB and IL-1β may amplify inflammation^15^ in the early stage of radiation proctitis. Vascular endothelial growth factor (VEGF) has a strong and specific effect on the growth of endothelial cells, and it is one of the most important cytokines to promote angiogenesis. Water channel proteins aquaporin-1 (AQP1) and aquaporin-3 (AQP3) may be involved in the angiogenesis and inflammation^16 - 18^ . Herein we sought to determine whether traditional Chinese medicine compound prescription Changrui enema has a role in the treatment of ARP by inhibiting inflammation-induced angiogenesis involved in regulation of IL-1β, NF-κB, VEGF, AQP1 and AQP3.

## Methods

### *Experimental animal*

Wild-type SPF mice (half male and half female, 5-7 weeks old, 18±2 grams weight) were purchased from Beijing Haidian Xingwang experimental animal farm (production license number: SCXK-2014-0013). All the mice were housed in the central laboratory of Shanxi Province Research Institute of Traditional Chinese Medicine for a week at 22±3°C and twelve-hour light/dark cycle in the laboratory. The mice were fed adaptively with standard laboratory chow (China Institute of Radiation Protection) for 1 week and were labeled by ear tags. The animal use protocol was reviewed and approved by the Institutional Animal Care and Use Committee (IACUC) of Shanxi Province Research Institute of Traditional Chinese Medicine (20150318).

### *Laboratory medicine and antibody*

Changrui enema (Sanguisorba officinalis L., Agrimonia pilosa Ledeb., Panax pseudoginseng, Bletilla striata, Colla corii asini, Rheum officinale Baill. and Acacia catechu) was purchased from Jiangyin Tianjiang Pharmaceutical Company Limited (production number: 0907129). Other mainly laboratory medicines included: dexamethasone sodium phosphate injection (Chenxin Pharmaceutical incorporated company, 1ml: 5mg, license number: NMPN H37021969), gentamicin sulphate injection (Henan Furen Huaiqing Tang Pharmaceutical Company Limited, 2ml: 8 million units, license number: NMPN41025466). Anti-IL-1β (sc-12742), anti-VEGF (sc-7269), anti-AQP1 (sc-25287), anti-AQP3 (sc-518001), anti-p-ERK1/2 (sc-7383), anti-ERK1/2 (sc-514302), anti-p-JNK (sc-6254), anti-JNK (sc-7345), anti-Bax (sc-7480) and anti-Bcl-2 (sc-7382) were purchased from Santa Cruz Biotechnology. Anti-NF-κB (AB16502) was purchased from ABCAM. DAB concentrated kits (PAB180021) was purchased from Bioswamp.

### *Acute radiation proctitis mice*

A total of 120 C57BL/6 mice were divided randomly into normal mice group (distilled water enema), ARP mice group (distilled water enema), western medicine enema group (dexamethasone 0.83mg/kg combined with gentamicin 13300U/kg), low-dose Changrui enema group (1800mg/kg), middle-dose Changrui enema group (3600mg/kg), and high-dose Changrui enema group (7200mg/kg); each group had 20 mice. ARP mice were established after 27Gy and 400cGy/min 6MV-X ray pelvic local irradiation by using linear accelerator (CX Series, VARIAN Company, USA) except for normal mice group.

### *Enema method*

On the second day, mice were treated by different enemas 1 time a day for 1 week or 2 weeks. These enemas were kept in the intestinal tract for 5 minutes. After treatment for 1 week or 2 weeks, 10 mice in each group were sacrificed after anesthesia and 1cm tissues of upper rectums were collected. Half tissue segments of rectums were stored in a refrigerator at −80°C for Western blot. The other tissue segments of rectums were fixed with paraffin embedded for H&E staining and immunohistochemistry staining.

### *Evaluation of rectal damage*

Based on previous studies^19^ , we proposed that the general signs of mice were scored as follows: (1) formed stool, nondestructive skin of the anus, normal diet and water intake, no weight loss, normal activity, smooth and clean hair; (2) unformed and mucous stool, diameter of perianal skin hair loss part was <0.5cm, diet and water intake decreased slightly, weight loss was about 1-2g, quiet activity, and smoothness of hair was slightly worse; (3) diarrhea, mucous bloody stool, diameter of perianal skin hair loss part was <0.5-1cm, diet and water intake decreased significantly, weight loss was 2-3g, curl up and less movement occasionally, and smoothness of hair was significantly worse; (4) diarrhea, watery stool and (or) archorrhagia, diameter of perianal skin hair loss part was >1cm, water repellent, antifeedant, weight loss >3g, curl up and less movement, stoop and writhe phenomenon, loss of smoothness of hair.

Considering that ARP often occurs during the first 2 weeks after irradiation, we observed the pathological changes of rectal tissue of ARP mice treated for 2 weeks. Pathological changes were graded as follows^20^: grade 0, normal rectal mucosa; grade 1, very slight damage, very slight rectal inflammation and (or) extremely slight damage of rectal gland; grade 2, slight damage, more obvious rectal inflammation or gland change than grade 1; grade 3, moderate damage, obvious rectal epithelial loss; and grade 4, serious damage, appear ulceration or necrosis.

### *Spleen index and liver index*

After treatment for 1 week or 2 weeks, the mice were anesthetized by intraperitoneal injection of pentobarbital sodium (50 mg/kg), then were sacrificed by cervical dislocation and decapitated. The bodies, livers and spleens of mice were removed and weighed. The liver index was calculated by the following formula: liver index (%) = liver wet weight / mouse body weight × 100%. The spleen index was calculated by the following formula: spleen index (%) = spleen wet weight / mouse body weight × 100%.

### *Absolute number of lymphocyte in spleen*

After treatment for 2 weeks, mice were sacrificed and spleen tissues were lysed by red blood cell lysis buffer and RIPA lysis buffer. The lymphocytes were isolated from spleen. The absolute number of lymphocytes in lymphocyte pellet suspension was counted by using haemocytometer three times. The absolute number of lymphocyte was calculated by the following formula: number of lymphocyte = number of cell mean/field of view × dilution factor (10) × 10^4^ /ml.

### *Immunohistochemistry*

Rectal tissues of mice were fixed by 4% formaldehyde, dehydrated, embedded in paraffin, sectioned and stained with H&E or immunohistochemistry. To block endogenous peroxidase activity, the section was blocked by 1% hydrogen peroxide in methanol for 10 minutes. Antigen was retrieved by microwave in 10 mM citrate buffer (pH 6.0) at 95°C for 10 minutes. Slides were blocked with goat serum for 20 minutes to reduce nonspecific binding. Then the sections were incubated in a humidified chamber at 4 °C overnight with one of the primary antibodies including anti- NF-κB (1:100), anti-VEGF (1:100), anti-AQP1 (1:100) and anti-AQP3 (1:100). The slides were washed three times with phosphate-buffered saline (PBS), incubated for 20 minutes with biotinylated second antibody, and labeled for 20 minutes with horseradish peroxidase. Peroxidase activity was visualized using a DAB concentrated kits. 3 fields of vision in each dyeing film were observed blindly by two experienced pathologists, respectively. The positive staining was quantitatively analyzed by image-Pro-plus software. The average optical density (AOD) = cumulative optical density (IOD) / total area (Area). These results were statistically analyzed by SPSS16.0.

### *Western blot*

Rectal tissues were lysed by RIPA lysis buffer and centrifuged at 4 °C at 13000 rpm for 20 minutes. Spleen tissues were lysed by red blood cell lysis buffer, then lysed by RIPA lysis buffer and centrifuged as above. Protein quantity was tested by the BCA protein assay. Then proteins were separated by SDS-PAGE and transferred to PVDF membrane. 5% skimmed milk was used to block nonspecific binding for 90 minutes. PVDF membrane was incubated overnight at 4 °C with primary antibodies such as anti-IL-1β (1:2000), anti-NF-κB (1:1000), anti-VEGF (1:1000), anti-AQP1 (1:1000), anti-AQP3 (1:1000), anti-p-ERK1/2 (1:2000), anti-ERK1/2 (1:1500), anti-p-JNK (1:1500), anti-JNK (1:1000), anti-Bax (1:1000) and anti-Bcl-2 (1:1000). Then PVDF membrane was washed by TBST (0.1%) and incubated with secondary antibodies. The specific protein bands were visualized by ECL chemiluminescence detection kit. The results were quantified by Quantity One software.

### *Statistical methods*

The data analyses were performed using statistical software SPSS16.0. Comparison of homogeneity of variance among multiple groups was performed using one-way analysis of variance (ANOVA). Multiple comparisons of heterogeneity variance were analyzed using Dunnett T3 method. The pathological grade was analyzed by Ridit analysis. *P* <0.05 was considered statistically significant.

## Results

### *Changrui enema treatment ameliorated general signs of ARP mice*

**Figure 1:**
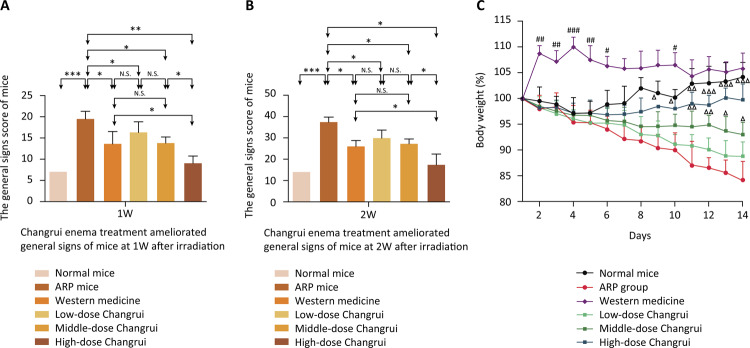
Changrui enema treatment ameliorated general signs of mice after irradiation. A. The difference in the general signs score of normal mice and ARP mice at 1 week was significant (*** *P* ﹤0.001). The general signs score was decreased in western medicine enema group and Changrui enema group compared with that of ARP mice at 1 week (* *P* ﹤0.05, ** *P* ﹤0.01). The high-dose Changrui enema treatment was more effective than western medicine enema treatment (* *P* ﹤0.05). B. The difference in the general signs score of normal mice and ARP mice at 2 weeks was significant (*** *P* ﹤0.001). The general signs score was decreased in western medicine enema group and Changrui enema group compared with that of ARP mice at 2 weeks (* *P* ﹤0.05). The high-dose Changrui enema treatment was more effective than western medicine enema treatment (* *P* ﹤0.05). C. The body weight change of ARP mice was decreased compared with that of normal mice at 8 to 14 days (* *P* ﹤0.05, ** *P* ﹤0.01, *** *P* ﹤0.001). Western medicine enema treatment increased body weight of mice compared with that of ARP mice at 2 to 6 days and 10 days (# *P* ﹤0.05, ## *P* ﹤0.01, ### *P* ﹤0.001). Changrui enema treatment also significantly restored body weight of ARP mice (Δ *P* ﹤0.05, ΔΔ *P* ﹤0.01, ΔΔΔ *P* ﹤0.001).

As shown in Figure 1 , there was no significant change in consumption food, water intake and body weight in normal mice. However, our results found that radiation reduced the mice food consumption and water intake, as well as inhibited gain in body weight. Some of the ARP mice showed a reduction of activity, piloerection, stooped on the second day. Some of the ARP mice began to defecate loose and bloody stools on the third day. There were 2 mice showed antifeedant, diarrhea, squint eye, unresponsive phenomenon and abdominal bulge on the fourth day. One of them was sacrificed and examined to show intestinal obstruction, and the other one died on the sixth day. The general signs score of ARP mice was higher than that of normal mice. Changrui enema and western medicine enema group (dexamethasone combined with gentamicin) were all effective in the treatment of ARP, and the high-dose Changrui enema was more effective than western medicine enema in the treatment of the general signs score of ARP mice ( Fig. 1 A-B ). The body weight of ARP mice was decreased; however, it was significantly restored by Changrui enema treatment and increased by western medicine enema treatment ( Fig. 1C ).

### *Changrui enema treatment reduced pathological grade of rectal tissue of ARP mice*

The results of pathological grade of rectal tissue of mice are shown in Table 2 . The differences between different groups were analyzed by ridit analysis. The pathological grade of rectal tissue of ARP mice was significantly higher than that of normal mice. The different treatments reduced the pathological grade of rectal tissue of ARP mice. There was no statistically significant difference between Changrui enema group and the western medicine enema group. These results suggested that the effect of Changrui enema in reducing the pathological grade of rectum was similar as the western medicine group. However, there was a trend for the better efficacy in high-dose Changrui enema group versus western medicine enema group according to ridit value.

**Table 2:** Comparison of pathological grades of rectal tissue under light microscopy of mice in different groups.

Pathological grade	Normal mice	ARP mice	Western medicine	Low-dose Changrui	Middle-dose Changrui	High-dose Changrui
Grade 0	10	0	0	0	0	1
Grade 1	0	0	2	1	3	4
Grade 2	0	1	4	5	5	5
Grade 3	0	4	3	4	2	0
Grade 4 Ridit value	0 0.0917	5 0.8433^***^	1 0.5925^#^	0 0.6017^#^	0 0.4967^##^	0 0.3742^###^

Compared to normal mice, *** *p* <0.001, compared to ARP mice, # *p* <0.05, ## *p* <0.01, ### *p* <0.05, ridit analysis

### *Changrui enema treatment did not affect spleen index and liver index*

**Figure 2:**
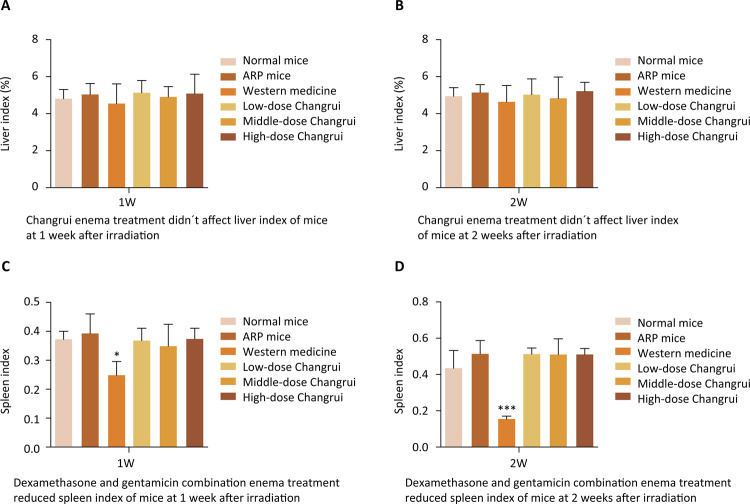
Changrui enema treatment did not affect spleen index and liver index. A. There was no significant difference in liver index at 1 week or 2 weeks between normal mice and ARP mice. Compared with ARP mice, liver index was not changed by Changrui enema treatment. B. The difference in liver index at 2 week between normal mice and ARP mice was also not significant. C. There was no significant difference in spleen index at 1 week between normal mice and ARP mice. Compared with ARP mice, spleen index was not changed by Changrui enema treatment. However, spleen index of mice was decreased by western medicine (* *P* < 0.05). D. The difference in spleen index at 2 week between normal mice and ARP mice was also not significant. Compared with ARP mice, spleen index was not changed by Changrui enema treatment. However, spleen index of mice was decreased by western medicine (*** *P* < 0.001), suggesting that the use of dexamethasone combined with gentamicin might reduce the weight of spleen and affect immunity responsiveness.

The indexes of spleen and liver reflect the immunity of mice at a certain extent. Liver is an important part of reticuloendothelial system, which is related to immunity. Spleen is the largest immune system organ in the body. The results showed that the pelvic local irradiation caused radiation proctitis, but did not affect the spleen index and liver index of mice. Western medicine (dexamethasone combined with gentamicin) reduced spleen index. Interestingly, Changrui enema did not affect spleen index and liver index compared with the normal mice ( Fig. 2 ).

### *IL-1β and NF-κB were down-regulated in the rectal tissues of ARP mice treated with Changrui enema*

**Figure 3:**
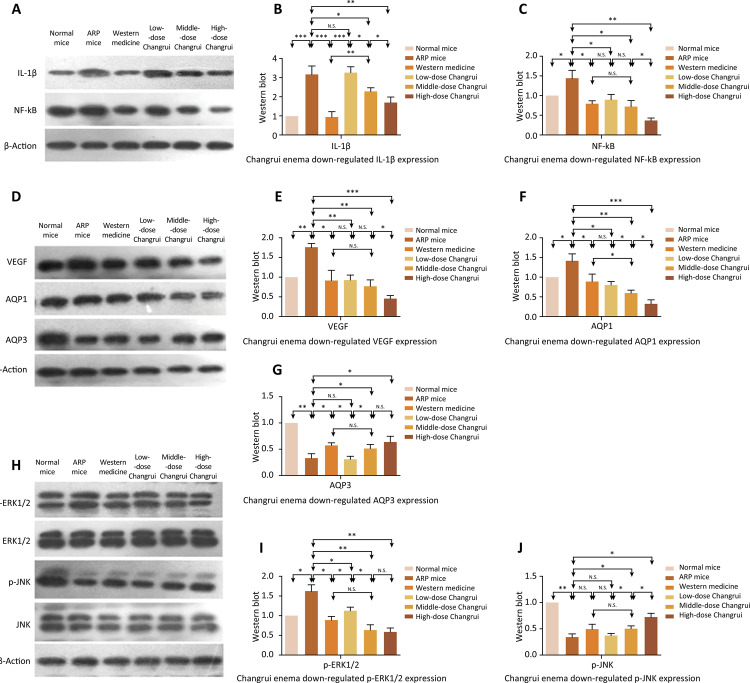
The expression of IL-1β, NF-κB, VEGF, AQP1, AQP3, p-ERK1/2 and p-JNK in different groups was tested by western blot. Western blot was performed to test the expression of IL-1β, NF-κB, VEGF, AQP1, AQP3, p-ERK1/2 and p-JNK in different groups (n=10 for each group). Compared to normal mice, the expression of IL-1β, NF-κB, VEGF, AQP1 and p-ERK1/2 was increased in ARP mice group and the expression of AQP3 and p-JNK was decreased in ARP mice group. Changrui enema and western medicine not only inhibited the expression of IL-1β, NF-κB, VEGF, AQP1 and p-ERK1/2, but also up-regulated the expression of AQP3 and p-JNK (* *P* <0.05, ** *P* <0.01, *** *P* <0.001).

**Figure 4:**
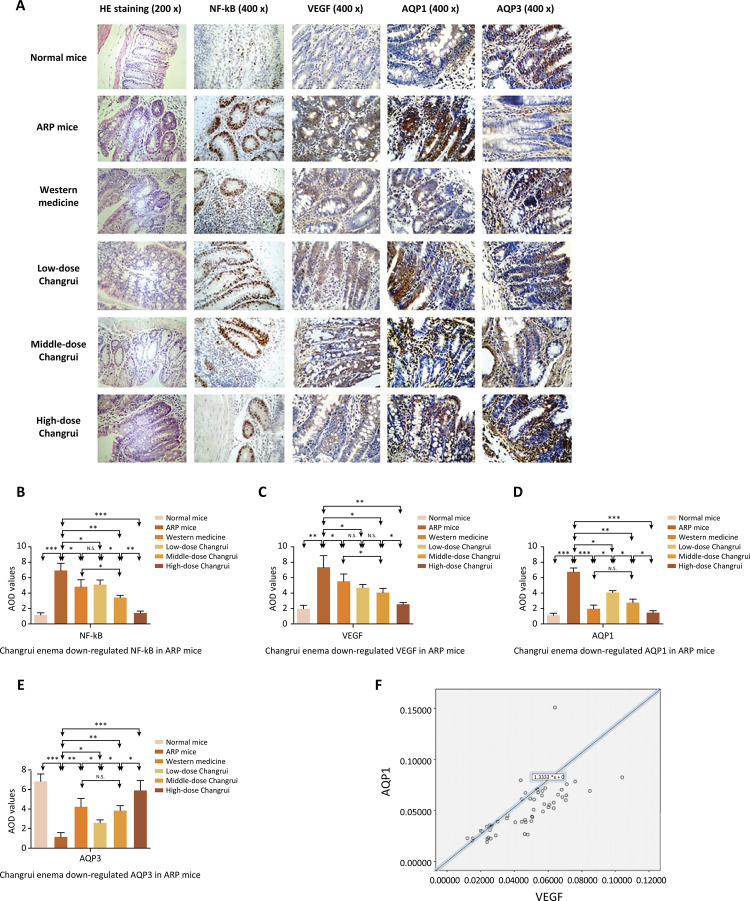
The immunohistochemistry imagines and AOD values of NF-κB, VEGF, AQP1 and AQP3 in different groups. A. HE stain and the immunohistochemistry of NF-κB, VEGF, AQP1 and AQP3 of the rectal tissues in different groups (n=10 for each group) were observed under light microscopy (Scale: ×400). The expression of NF-κB (B), VEGF (C) and AQP1 (D) was increased in ARP mice group and was inhibited by Changrui enema and western medicine. E. The expression of AQP3 was decreased in ARP mice group and was up-regulated by Changrui enema and western medicine (* *P* <0.05, ** *P* <0.01, *** *P* <0.001). F. The result showed that there was a positive correlation between VEGF and AQP1.

The western blot results of each group are shown in Figure 3 A-C. The expression of IL-1β and NF-κB in rectal tissue of ARP mice was significantly higher than those of normal mice. The expression of IL-1β and NF-κB in the different treatment group was lower than those of ARP group. Furthermore, we found that a high dose Changrui enema was the most effective in inhibiting the expression of NF-κB of ARP mice. The immunohistochemistry results of each group are shown in Figure 4A . Middle-dose and high-dose Changrui enema were more effective than western medicine (dexamethasone combined with gentamicin) in inhibiting the expression of NF-κB of ARP mice ( Fig. 4B ).

### *VEGF and AQP1 were down-regulated and AQP3 was up-regulated by Changrui enema treatment in the mice rectal tissues*

As shown in Figure 3 D-G and Figure 4A, 4C-E, the expression of VEGF and AQP1 in rectal tissue of mice in ARP mice group was increased significantly compared to normal mice group. However, the expression of AQP3 in rectal tissue of mice in ARP mice group was decreased significantly compared to normal mice group. Interestingly, the enema treatment of Changrui and dexamethasone combined with gentamicin down-regulated VEGF and AQP1, while up-regulated AQP3. These results suggested that the effect of AQP3 was contrary to the effect of AQP1 in the development of radiation proctitis.

### *P-ERK1/2 was down-regulated and p-JNK was up-regulated by Changrui enema treatment in the mice rectal tissues*

As shown in Figure 3 H-J, the expression of p-ERK1/2 in rectal tissue of mice in ARP mice group was increased significantly compared to normal mice group. In contrast to AQP1, the expression of AQP3 in rectal tissue of mice in ARP mice group was decreased significantly compared to normal mice group. Furthermore, the enema treatment of Changrui and dexamethasone combined with gentamicin down-regulated p-ERK1/2, while up-regulated p-JNK. These results suggested that Changrui might inhibit AQP1 expression by down-regulating p-ERK1/2, and improve AQP3 expression by up-regulating p-JNK.

### *Dexamethasone combined with gentamicin enema reduced lymphocyte number in spleen by increasing Bax expression and decreasing Bcl-2 expression*

**Figure 5:**
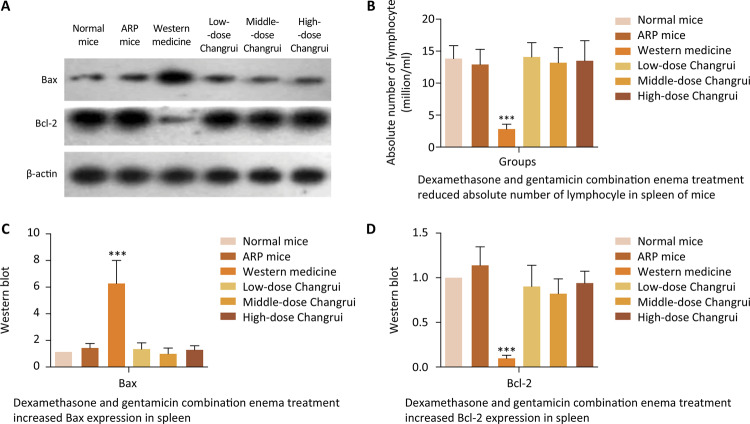
Absolute number of lymphocyte and the expression of Bax and Bcl-2 in spleen in different groups were observed and tested. A. Western blot was performed to test the expression of Bax and Bcl-2 in spleen in different groups (n=10 for each group). B. Absolute number of lymphocyte was reduced in spleen of mice treated with dexamethasone and gentamicin combination enema for 2 weeks (*** *P* <0.001). C. Compared to normal mice, the expression of Bax was increased in spleen tissue of mice treated by dexamethasone combined with gentamicin (*** *P* <0.001). D. The expression of Bcl-2 was decreased in spleen tissue of mice treated by dexamethasone combined with gentamicin (*** *P* <0.001).

As shown in Figure 5B , absolute number of lymphocyte was reduced in spleen of mice treated with dexamethasone and gentamicin combination enema for 2 weeks. There was no significant difference in absolute number of lymphocyte among Changrui enema group, normal mice group and RP mice group. Absolute number of lymphocyte was not affected by Changrui enema treatment as well as by pelvic local irradiation. The mechanism related with apoptosis was studied by testing Bax and Bcl-2 expression in spleen ( Fig. 5A ). Bax expression was increased significantly while Bcl-2 expression was decreased in spleen tissue of mice treated by dexamethasone combined with gentamicin ( Fig. 5 A,C,D ). But Changrui enema treatment and pelvic local irradiation did not affect Bax and Bcl-2 expression ( Fig. 5 A,C ). These results suggested that dexamethasone combined with gentamicin might reduce spleen weight and lymphocyte number in spleen by increasing Bax expression and decreasing Bcl-2 expression.

## Discussion

Radiation proctitis is one of the most common complications of abdominal and pelvic radiation. There are various treatments for radiation proctitis and these include medical therapies, hyperbaric oxygen and endoscopic therapies^20^ . Many medical therapies have been proposed and developed, including anti-inflammatory agents (sulphasalazine, balsalazide and mesalazine), antioxidants (vitamin A, E and C)^21 , 22^ , mucosal protectants (formalin, sildenafil and mesenchymal stem cell)^23 , 24^ and so on. Apart from medical therapies, hyperbaric Oxygen appears to be an effective therapy for radiation proctitis according to some clinical trials^25^ . In addition, endoscopic therapies have also been developed for the treatment of radiation proctitis, including laser therapies^26 , 27^ , argon plasma coagulation^28 , 29^ , radiofrequency ablation^30 , 31^ and cryoablation^32 , 33^ . However, these therapies of radiation proctitis still remain unsatisfactory and novel treatments are urgently needed. Available literature^34^ has found that TCM may have a great potential effect in the treatment of radiation proctitis. A preliminary randomized controlled clinical trial found that Aloe vera was a safe and effective therapy for ARP. Aloe vera not only relieved diarrhea and fecal urgency scores, but also improved the quality of life of ARP patients^35^ . We found that retention enema with TCM compound prescription Changrui enema was an effective treatment of ARP in clinical application.

ARP is characterized by bloody and diarrhea, which indicate that inflammation and angiogenesis are involved in ARP^36^ . We proposed that inflammation and angiogenesis occurred in early stage and late stage, respectively. Radiation induces IL-1β, which activates NF-κB^37^ and other inflammatory factors. Conversely, NF-κB promotes the transcription of IL-1β^38^ . NF-κB and IL-1β form a positive feedback loop, which further amplifies inflammation in the early stage of ARP. Furthermore, we found that severe congestion of rectal mucosa occurred in late stage of ARP in previous clinical trials. Angiogenesis may participate in the development of ARP. As we all know, VEGF is one of the most important angiogenesis factors^39^ . Therefore, we assumed that inflammation-induced angiogenesis played a key role in ARP. This hypothesis is supported by some studies of other diseases. Key transcription factors involved in inflammatory and angiogenesis, NF-κB as well as HIF-1 and other cytokines are mutually regulated^40^ . Selective inhibition of non-canonical NF-κB signaling may be beneficial not only in chronic inflammatory diseases but also in other diseases characterized by aberrant neovascularization^41^ . NF-κB is involved in the upregulation of VEGF mRNA and inhibition of NF-κB activity decreases the VEGF mRNA in breast cancer cell^42^ . These findings suggest that NF-κB regulates VEGF at the transcriptional level.

Our study also found that the high expression of NF-κB was accompanied by increased VEGF in rectal tissue of ARP mice. Dexamethasone combined with gentamicin inhibited the expression of VEGF by down-regulating NF-κB in rectal tissue of ARP mice. Unfortunately, dexamethasone combined with gentamicin led to reduction of spleen weight. Spleen is the largest lymphoid organ, which modulates the immune system by producing lymphocytes, immunoglobulin, complement and other immune substances^43^ . Dexamethasone combined with gentamicin reduced spleen weight and lymphocyte number in spleen by inducing lymphocyte apoptosis. The spleen cell apoptosis might be regulated by increase of Bax expression and decrease of Bcl-2 expression. Changrui enema significantly inhibited the expression of VEGF mediated by NF-κB in rectal tissue of ARP mice, but did not affect the spleen weight and lymphocyte number in spleen. Contrary to dexamethasone combined with gentamicin, Changrui enema did not influence spleen-mediated immunity. And High-dose Changrui enema had more effective in inhibiting NF-κB, VEGF and AQP1 compared to dexamethasone combined with gentamicin and other treatments.

Angiogenesis is a complex process and regulated by many kinds of signaling factors. In addition to the proliferation of endothelial cells stimulated by VEGF, the migration of endothelial cells is also a crucial factor in angiogenesis. AQP1 promotes the migration of endothelial cells, and is involved in angiogenesis of tumor^44 - 47^ . We examined the expression of AQP1 and VEGF in rectal tissues of radiation proctitis mice by immunohistochemistry and western blot, and found that there was a positive correlation between them. A study has found that AQP3 expression was decreased in intestinal inflammation induced by trinitrobenzene sulfonic acid, which resulted in reduction of intestinal water re-absorption and diarrhea symptoms^48^ . We proposed that AQP3 might suppress intestinal inflammation. Our results showed that AQP3 expression in ARP mice was significantly decreased compared to that in normal mice. We speculate AQP1 promotes angiogenesis, while AQP3 inhibits inflammation. ERK MAPK pathway has been verified to correlate with angiogenesis^49^ and regulates AQP1 in a hypoxia model of Schwann cells in vitro^50^ . JNK pathway is involved in the regulation of AQP3 in isolated oligohydramnios tissues^51^ . We found that the expression of p-ERK1/2 was increased in rectal tissue of ARP mice. In contrast to p-ERK1/2, the expression of p-JNK was decreased significantly in ARP mice. Excitedly, p-ERK1/2 was down-regulated, while p-JNK was up-regulated by Changrui as well as dexamethasone combined with gentamicin. Although the precise mechanism needs to be further explored, our findings suggest that Changrui maybe inhibits AQP1 expression by down-regulating p-ERK1/2, and improves AQP3 expression by up-regulating p-JNK.

## Conclusions

Our study firstly found that TCM compound prescription Changrui enema alleviated ARP mice. Changrui enema decreased general signs score and rectal tissue pathological grades of radiation proctitis in mice. The mechanism of Changrui enema might be related with the inhibition of inflammation-induced angiogenesis. Changrui not only inhibited inflammation factors IL-1β and NF-κB and angiogenesis factor VEGF, but also increased anti-inflammation factor AQP3 and decreased angiogenesis related factor AQP1. Together with these results, this study innovates the pathogenesis of ARP and provides a new effective treatment of ARP by using enema of TCM. However, some questions remain unanswered, for example we do not know the specific interaction between aquaporin and MAPK signaling pathways. Further studies should be carried out to investigate the molecular mechanism involved in AQP1, p-ERK1/2, AQP3 and p-JNK.
